# The *Aspergillus giganteus *antifungal protein AFP_NN5353 _activates the cell wall integrity pathway and perturbs calcium homeostasis

**DOI:** 10.1186/1471-2180-11-209

**Published:** 2011-09-23

**Authors:** Ulrike Binder, Mojca Bencina, Andrea Eigentler, Vera Meyer, Florentine Marx

**Affiliations:** 1 Biocenter, Division of Molecular Biology, Innsbruck Medical University, Fritz-Pregl Strasse 3, Innsbruck, A-6020, Austria; 2 Department of Biotechnology, National Institute of Chemistry, Hajdrihova 19, Ljubljana, SI-1000, Slovenia; 3 Excellent NMR, Future Innovation for Sustainable Technologies Centre of Excellence, Hajdrihova 19, Ljubljana, SI-1000, Slovenia; 4 Department of Applied and Molecular Microbiology, Institute of Biotechnology, Berlin University of Technology, Gustav-Meyer-Allee 25, Berlin, D-13355, Germany; 5 Department of Hygiene, Microbiology and Social Medicine, Innsbruck Medical University, Fritz-Pregl Strasse 3, Innsbruck, A-6020, Austria

## Abstract

**Background:**

The antifungal protein AFP_NN5353 _is a defensin-like protein of *Aspergillus giganteus*. It belongs to a group of secretory proteins with low molecular mass, cationic character and a high content of cysteine residues. The protein inhibits the germination and growth of filamentous ascomycetes, including important human and plant pathogens and the model organsims *Aspergillus nidulans *and *Aspergillus niger*.

**Results:**

We determined an AFP_NN5353 _hypersensitive phenotype of non-functional *A. nidulans *mutants in the protein kinase C (Pkc)/mitogen-activated protein kinase (Mpk) signalling pathway and the induction of the α-glucan synthase A (*agsA*) promoter in a transgenic *A. niger *strain which point at the activation of the cell wall integrity pathway (CWIP) and the remodelling of the cell wall in response to AFP_NN5353_. The activation of the CWIP by AFP_NN5353_, however, operates independently from RhoA which is the central regulator of CWIP signal transduction in fungi.

Furthermore, we provide evidence that calcium (Ca^2+^) signalling plays an important role in the mechanistic function of this antifungal protein. AFP_NN5353 _increased about 2-fold the cytosolic free Ca^2+ ^([Ca^2+^]_c_) of a transgenic *A. niger *strain expressing codon optimized aequorin. Supplementation of the growth medium with CaCl_2 _counteracted AFP_NN5353 _toxicity, ameliorated the perturbation of the [Ca^2+^]_c _resting level and prevented protein uptake into *Aspergillus sp*. cells.

**Conclusions:**

The present study contributes new insights into the molecular mechanisms of action of the *A. giganteus *antifungal protein AFP_NN5353_. We identified its antifungal activity, initiated the investigation of pathways that determine protein toxicity, namely the CWIP and the Ca^2+ ^signalling cascade, and studied in detail the cellular uptake mechanism in sensitive target fungi. This knowledge contributes to define new potential targets for the development of novel antifungal strategies to prevent and combat infections of filamentous fungi which have severe negative impact in medicine and agriculture.

## Background

All organisms have evolved several defence systems in order to protect themselves against bacteria, fungi and viruses. Higher organisms have developed a complex network of humoral and cellular responses, called adaptive immunity. A second defence system, the innate immunity, consists of many components, including small peptides with a broad antimicrobial spectrum [1,2]. The production of such proteins with antimicrobial activity is not limited to higher eukaryotes, but also found in microorganisms, including fungi. The diversity of these proteins is reflected in their mode of action and their species-specificity. Some of them form pores in the membrane, others are known to inhibit cell wall synthesis or interfere with nucleic acids and their synthesis [3,4]. They can be involved in the inhibition of protein synthesis or interfere with cell cycle control [3,4]. A relatively new group of antimicrobial proteins secreted by filamentous ascomycetes includes small, cationic and cysteine-rich proteins. So far, only few antifungal proteins have been characterized, namely AFP from *Aspergillus giganteus*, ANAFP from *Aspergillus niger*, PAF from *Penicillium chrysogenum *and NAF from *Penicillium nalgiovense *[5-8].

The mode of action of these proteins is not fully understood. Nevertheless, there is evidence, that their toxicity is mediated by interaction with distinct molecules or receptors at the outer layers of the cell, e.g. cell wall or plasma membrane. Deleterious effects can then be induced either by transmitting signals from the outer layers into the cell, or by internalization of the protein and interaction with internal molecules [9-15]. Similar to substances that perturb the cell wall, such as caspofungin, congo red or calcofluor white (CFW) [10,16], the *A. giganteus *antifungal protein AFP was found to modulate the cell wall composition by enhancing the expression of the α-1,3-glucan synthase A gene (*agsA*), possibly by the activation of the cell wall integrity pathway (CWIP), and inhibiting chitin synthesis in sensitive fungi [10]. This, however, stands in contrast to the mode of action of the *P. chrysogenum *antifungal protein PAF which fails to activate the CWIP [9]. However, the central players that trigger cell wall remodelling in AFP-sensitive fungi have not been investigated so far.

Another mechanistic function of antifungal proteins is the interference with ion, especially Ca^2+ ^ion homeostasis and signalling [15,17,18]. We could recently show that the *P. chrysogenum *antifungal protein PAF severely perturbed the Ca^2+ ^homeostasis of *Neurospora crassa *by rapidly elevating the cytoplasmic Ca^2+ ^[Ca^2+^]_c _resting level [17]. Numerous reports indicate that the activity of antifungal proteins can be antagonized by the external addition of Ca^2+ ^ions to the test medium [15,17-21] pointing towards the induction of adaptive responses which may be triggered by Ca^2+ ^signalling [15,17].

The aim of this study was to characterize in more detail the mode of action of the *A. giganteus *AFP variant protein AFP_NN5353 _and to investigate the pathways that might be affected/modulated by this antifungal protein. Therefore, we focussed our interest on the involvement of the CWIP and the Ca^2+ ^signalling in the toxicity of AFP_NN5353_. To address these questions, we used the highly AFP_NN5353 _sensitive model organisms *A. nidulans *and *A. niger *for which appropriate mutant strains were available.

## Results

### In silico analysis of AFP_NN5353_

**Figure 1:**
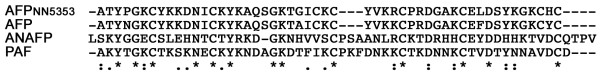
**Clustalw sequence alignment http://www.ebi.ac.uk/Tools/msa/clustalw2/ of the antifungal proteins AFP_NN5353 _and AFP from *A. giganteus*, ANAFP from *A. niger *and PAF from *P. chrysogenum***. Identical amino acids (aa) are marked with (*), aa with strong similarity are indicated with (:) and aa with weak similarity are marked with (.).

CLUSTALW amino acid (aa) sequence analysis of AFP_NN5353 _with other known antifungal proteins revealed that AFP_NN5353 _from *A. giganteus *strain A3274 is a protein homologous to AFP from *A. giganteus *strain MDH 18894 [8,22]. AFP_NN5353 _exhibits > 90% identity with AFP, but only 42% identity with the *P. chrysogenum *PAF and 27% identity with the *A. niger *ANAFP. In fact, the secreted mature form of AFP_NN5353 _consists of 51 aa and differs only in 5 aa from AFP (Figure 1). Three aa exchanges belong to structurally related aa, one aa exhibits weak similarity and one aa is different (position 4). These aa exchanges do not influence the theoretical isoelectric point (pI) of AFP_NN5353_, which is the same as for AFP (pI 9.3, http://expasy.org/tools/protparam.html). Most importantly, AFP_NN5353 _still contains the putative chitin-binding domain CKYKAQ present in AFP but not in PAF or ANAFP and also harbors all conserved cysteine residues important for protein stabilization [10,23].

### Antifungal activity of the protein AFP_NN5353_

To investigate the antifungal specificity of AFP_NN5353_, fifteen filamentous fungi were tested for their susceptibility to the protein. Since antifungal proteins might be useful for biotechnological applications, filamentous human and plant pathogenic fungi were selected as test organisms (e.g. *Fusarium oxysporum*, *Botrytis cinerea*, *Mucor *sp. and *A. fumigatus*) in addition to the model organisms *A. nidulans *and *A. niger*. As shown in Table 1, thirteen out of fifteen tested moulds were found to be sensitive against AFP_NN5353_. *A. nidulans *wild type, *N. crassa *wild type and *A. niger *wild type were the most sensitive strains to AFP_NN5353_. The minimal inhibitory concentration (MIC) of AFP (the concentration that completely inhibited conidial germination in liquid growth assays) was 0.2 μg/ml for *A. nidulans*, 0.5 μg/ml for *N. crassa *and 1 μg/ml for *A. niger*. Two strains were unaffected at the protein concentrations tested: *M. circenelloides *and *M. genevensis *were insensitive against AFP_NN5353 _when concentrations up to 500 μg/ml were used.

**Table 1:** Minimal inhibitory concentrations (MIC; μg/ml) of AFP_NN5353 _against different filamentous fungi.

organism	MIC (μg/ml)
*Aspergillus flavus *ATCC9643	50
*Aspergillus fumigatus *ATCC 46645	50
*Aspergillus giganteus *AG090701	50
*Aspergillus nidulans *FGSC4	0.2
*Aspergillus niger *CBS 120.49	1
*Aspergillus terreus *304	5
*Botrytis cinerea *BC 080801	10
*Fusarium oxysporum *FO 240901	5
*Fusarium sambucinum *FS210901	5
*Gliocladium roseum *GR 210901	100
*Mucor circinelloides *MC080801	insensitive^a^
*Mucor genevensis *MG 080801	insensitive^a^
*Penicillium chrysogenum *ATCC10002	10
*Trichoderma koningii *TC 060901	20
*Neuropsora crassa *FGSC 2489	0.5

^a^up to 500 μg/ml AFP_NN5353 _was tested

1 × 10^4 ^conidia/ml were incubated in 200 μl CM medium in the presence of various concentrations of AFP_NN5353 _at 30°C for 24 h. Growth was determined by measuring the OD_620 nm_.

### AFP_NN5353 _interferes with the cell wall integrity of *A. nidulans*

It is known that antifungal compounds such as congo red, caffeine, CFW or caspofungin interfere with cell wall biosynthesis and weaken the cell wall in fungi (reviewed by [24]). The remodeling of the cell wall by these antifungal compounds is mediated by the activation of the CWIP. In fungi, extracellular signals are transmitted via the membrane bound small GTPase RhoA to the central regulators Pkc and Mpk, which are regulated by phosphorylation/dephosphorylation. The signal transduction cascade eventually enforces transcription of cell wall synthesis genes, partly via the transcription factor RlmA [16,25]. Respective loss-of-function or conditional mutants show hypersensitive phenotypes in the presence of cell wall perturbing agents [9,24-26]. Similar to substances that weaken the cell wall, the *A. giganteus *antifungal protein AFP modulates the cell wall composition by inhibiting chitin synthesis in sensitive fungi (e.g. *A. niger*, *A. oryzae*) and inducing the expression of *agsA *most likely by the activation of the CWIP [10].

To study the involvement of the CWIP in AFP_NN5353 _toxicity, we first tested whether the osmotic stabilizer sorbitol counteracts the toxicity of AFP_NN5353_. In the absence of AFP_NN5353 _*A. nidulans *proliferated less well in the presence of 1 M sorbitol and reached only 30% growth compared to the growth in standard medium (100%). Nevertheless, the addition of 1 M sorbitol to the growth medium strongly reduced the activity of AFP_NN5353 _on *A. nidulans *wild type. The osmotic stabilizer ameliorated growth in the presence of 0.05 μg/ml AFP_NN5353 _by 80% compared to a 10% growth rate in the absence of sorbitol (Table 2). This was even more accentuated when 0.1 and 0.2 μg/ml AFP_NN5353 _were applied, suggesting that AFP_NN5353 _indeed weakens the cell wall of *A. nidulans*.

**Table 2:** The effect of 1 M sorbitol on the growth inhibiting activity of AFP_NN5353 _on *A. nidulans*.

AFP_NN5353 _(μg/ml)	CM	CM + 1 M sorbitol
0	100 (_SD _± 10)	100 (_SD _± 11)
0.05	10.4 (_SD _± 1)	79.3 (_SD _± 6)
0.1	5.5 (_SD _± 2)	68.3 (_SD _± 0.8)
0.2	no growth	17.8 (_SD _± 0.8)

1 × 10^4 ^conidia/ml were incubated in CM with 0-0.2 μg/ml AFP_NN5353 _for 24 h. Percent values were calculated from percent changes in OD_620 _of AFP_NN5353 _treated *A. nidulans *compared to untreated controls (= 100%). Results are expressed as mean ± SD (n = 3).

To investigate whether AFP_NN5353 _induces *agsA *gene transcription similar to AFP via the Pkc/Mpk signalling pathway, we tested the effect of the antifungal protein on the transgenic *A. niger *strain RD6.47 which expresses a nuclear-targeted GFP protein fused to the *A. niger agsA *promoter. RD6.47 germlings were treated with AFP_NN5353 _(conc. 10 to 100 μg/ml) for 2 h and analyzed microscopically. As shown in Additional file 1, a nuclear signal was clearly detectable in germlings of RD6.47 treated with ≥ 50 μg/ml AFP_NN5353_, similar to that when exposed to 10 μg/ml caspofungin. In untreated germlings, however, no signal could be observed. These observations perfectly match with the data obtained for AFP [10]. It has to be noted here that antifungal protein concentrations higher than the MIC determined for conidia (> 10-50 fold) are needed to inhibit the growth of germlings or hyphae of sensitive fungi [10,27] (data not shown).

**Figure 2:**
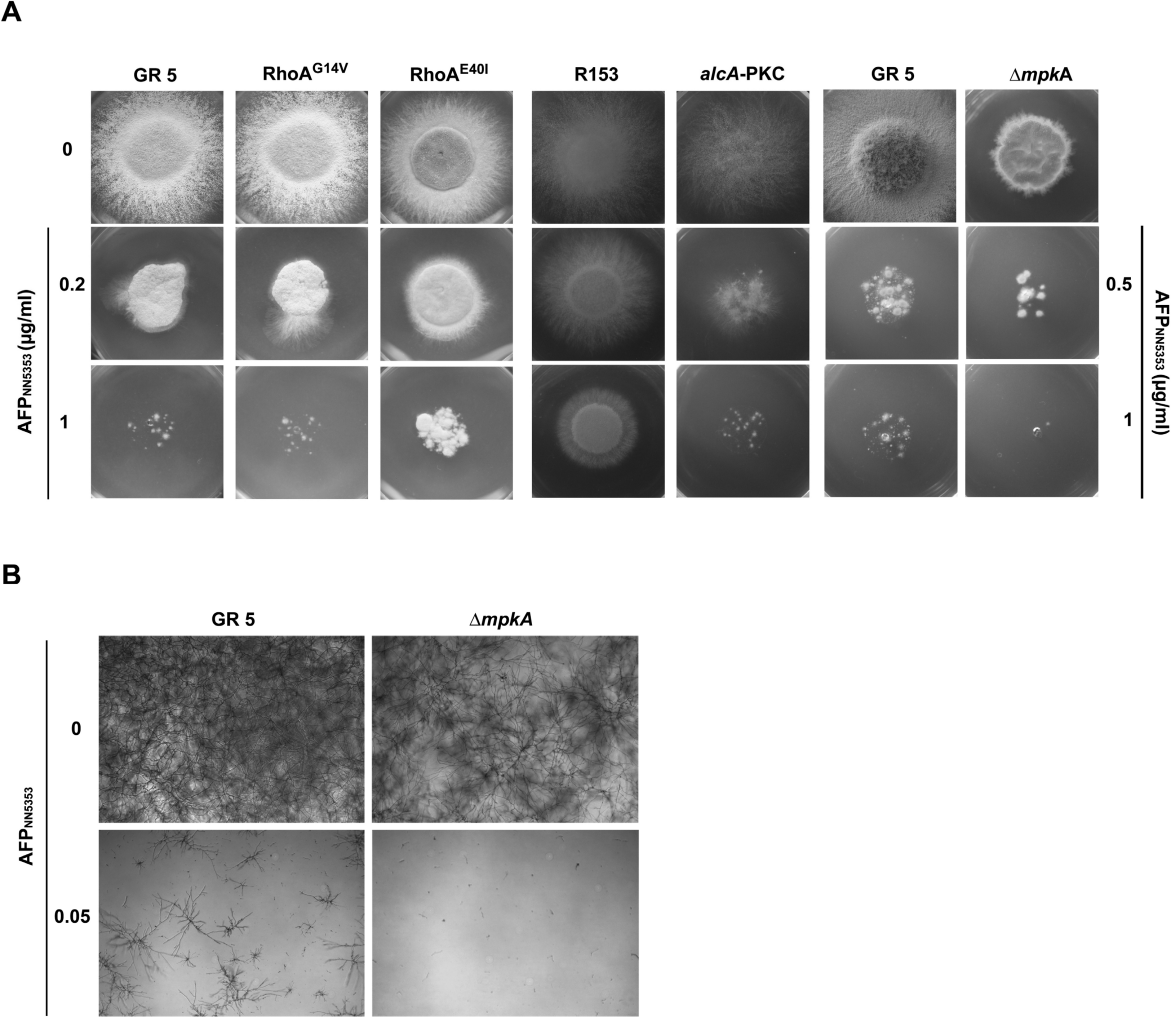
**AFP_NN5353 _susceptibility of *A. nidulans *mutants RhoA^G14V^, RhoA^E40I^, *alcA*-PkcA and Δ*mpkA *compared to the respective recipient strains GR5 and R153**. **(A) **A total of 2 × 10^3 ^conidia were point inoculated on agar plates (CM for GR5, RhoA^G14V^, RhoA^E40I ^and Δ*mpkA*, repressive MM containing 1% glucose according to [26] for R135 and *alcA*-PkcA) containing the appropriate supplements and 0, 0.2 and 1 μg/ml AFP_NN5353 _for GR5, RhoA^G14V^, RhoA^E40I^, R135 and *alcA*-PkcA. The Δ*mpkA *mutant and its reference strain GR5 were exposed to 0, 0.5 and 1 μg/ml AFP_NN5353_. The plates were incubated at 37°C for 48 h. **(B) **1 × 10^4 ^conidia/ml of the Δ*mpkA *mutant and GR5 were treated with 0.05 μg/ml AFP_NN5353 _or without protein (controls) in a total volume of 200 μl of appropriately supplemented CM in 96-well plates.

Next, we tested several *A. nidulans *mutant strains affected in central players of the CWIP for their susceptibility to AFP_NN5353 _by determining their radial growth in the presence or absence of the antifungal protein. Since RhoA is an essential protein in *A. nidulans*, two strains with ectopic copies of the constitutively active *rhoA^G14V ^*allele and the dominant *rhoA^E40I ^*allele [28] were tested in comparison to the wild type strain (GR5). The *rhoA*^G14V ^mutation prevents the hydrolysis of GTP and therefore renders RhoA constantly active [28]. Similarly, the GTP hydrolysis is inhibited in the RhoA^E40I ^strain, but this mutation also perturbs the binding of the GTPase activating protein (GAP) to RhoA and possibly disturbs downstream effectors of RhoA-GAP [28]. The constitutively active RhoA^G14V ^and the dominant RhoA^E40I ^strain exhibited the same sensitivity towards AFP_NN5353 _as the wild type strain at low protein concentrations (≤ 0.2 μg/ml) (Figure 2A). Interestingly, the dominant RhoA^E40I ^strain was more resistant to AFP_NN5353 _than the wild type strain or the RhoA^G14V ^strain at higher protein concentrations (1 μg/ml) (Figure 2A). Therefore, we suggest that the toxicity of AFP_NN5353 _is transmitted by RhoA-GAP targets and not by RhoA itself. These mutants performed similarly when exposed to the orthologous *P. chrysogenum *antifungal protein PAF [9].

In addition, mutants defective in PkcA and MpkA activity were tested for their AFP_NN5353 _susceptibility. As *pkcA *is an essential gene in *A. nidulans*, a conditional *alcA*-PKC mutant strain was used, where the *pkcA *gene was put under the control of the *alcA *promoter, which is repressed by glucose but derepressed by glycerol [26]. Both the conditional *alcA*-PKC mutant (cultivated under repressive conditions) and a Δ*mpkA *mutant were hypersensitive to AFP_NN5353 _compared to their recipient strains R153 and GR5, respectively, indicating that the activity of PkcA and MpkA confers a certain resistance to AFP_NN5353 _(Figure 2A). The hypersensitive phenotype of the Δ*mpkA *mutant was also confirmed by liquid growth inhibitory assays. In unchallenged liquid condition, the GR5 and the Δ*mpkA *mutant showed a comparable proliferation rate (Figure 2B). In the presence of 0.05 μg/ml AFP_NN5353_, however, the *mpkA *deletion strain did not germinate whereas the GR5 strain still exhibited 11% growth. Note that growth inhibition in liquid conditions requires less antifungal protein to monitor its toxicity than on solid media probably due to less diffusion in the latter case (data not shown).

From these data we conclude that AFP_NN5353 _interferes with the cell wall homeostasis of *A. nidulans *and that this interaction is mediated by PkcA/MpkA signalling, although independently from RhoA.

### AFP_NN5353 _disrupts calcium homeostasis in *A. niger*

Supplements other than osmotic stabilizers can also antagonize the activity of antifungal proteins from plants and ascomycetes. For example, the addition of cations such as Ca^2+ ^ions to the growth medium reversed the antifungal activity of the *P. chrysogenum *PAF [17], the *A. giganteus *AFP [15,21] and of plant defensins [29,30] which are usually positively charged due to their high pI. A cation-sensitive antifungal mode of action can for example be associated with the perturbation of the intracellular Ca^2+ ^homeostasis by antifungal peptides [17,18] but might also result from the interference of cations with antifungal-target interaction(s).

Therefore, we tested to which extend these effects also account for the antifungal activity of AFP_NN5353_. To this end, we selected *A. niger *as model organism because this mould was highly sensitive to AFP_NN5353 _and a transgenic strain was available that expressed the recombinant codon optimized Ca^2+^-sensitive photoprotein aequorin for measuring the [Ca^2+^]_c _resting level in response to AFP_NN5353 _[31]. First, we tested the activity of AFP_NN5353 _in Vogels* medium supplemented with 5-20 mM CaCl_2 _or without CaCl_2 _as a control (data not shown). Addition of CaCl_2 _did not influence the growth of *A. niger *up to a concentration of 20 mM. The growth of *A. niger *exposed to AFP_NN5353_, however, ameliorated in the presence of increasing concentrations of CaCl_2_. 20 mM CaCl_2 _neutralized the toxicity of 0.5-1.0 μg/ml AFP_NN5353 _and the treated samples resumed growth to 100% (Table 3).

**Table 3:** The effect of 20 mM external CaCl_2 _(in Vogels* medium) on the growth inhibitory activity of AFP_NN5353 _on *A. niger *strain A533.

AFP_NN5353 _(μg/ml)	Vogels*	Vogels* + 20 mM Ca^**2+**^
0	100 (_SD _± 10)	100 (_SD _± 8)
0.5	12 (_SD _± 3)	101 (_SD _± 9)
1.0	no growth	105 (_SD _± 6)

OD_620 _was measured after 24 h of incubation. The growth of untreated controls was normalized to 100% to evaluate the percent growth of samples in the presence of AFP_NN5353_. Vogels* medium without CaCl_2 _supplementation contains 0.7 mM Ca^2+^. Results are expressed as mean ± SD (n = 3).

**Figure 3:**
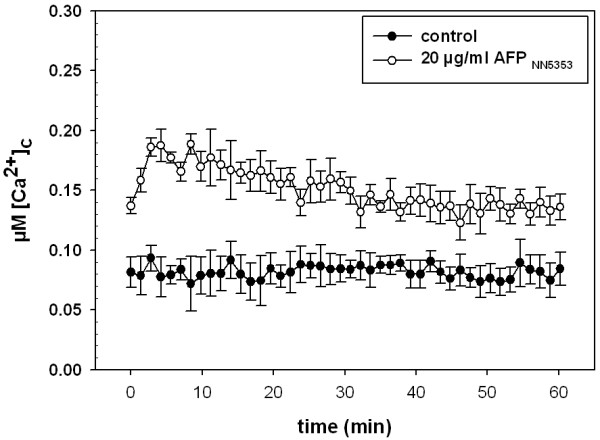
**Increase in resting [Ca^2+^]_c _of twelve h old *A. niger *germlings treated with AFP_NN5353 _or no protein (controls)**. Measurements were taken every 1.4 minutes. Values represent average of six samples.

Next, we determined the influence of AFP_NN5353 _on the intracellular Ca^2+ ^signature. Before AFP_NN5353 _addition, the resting level of the intracellular Ca^2+ ^was 0.08 μM. We could show, however, that the [Ca^2+^]_c _resting level was significantly increased in twelve h old *A. niger *cultures that were treated with 20 μg/ml AFP_NN5353_. The [Ca^2+^]_c _resting level rose to a maximum of 0.19 μM within the first 8 min and stayed elevated throughout the time of measurement (60 min), whereas the Ca^2+ ^level of the untreated control remained at 0.08 μM (Figure 3). This indicated that AFP_NN5353 _indeed disrupts Ca^2+ ^homeostasis in *A. niger*.

To exclude the possibility that the AFP_NN5353 _induced rise in the [Ca^2+^]_c _resting level is due to membrane permeabilization and/or pore formation, we studied the effects of AFP_NN5353 _on germlings in the presence of CMFDA, a membrane permeant dye that is metabolized by viable cells, and the membrane impermeant dye propidium iodide (PI). Additional file 2 shows that samples treated with 20 μg/ml AFP_NN5353 _for 10 min metabolized CMFDA but did not take up PI, resulting in green but no red fluorescence, similar to untreated controls. This indicated that the plasma membrane was still intact after 10 min of protein treatment. Samples exposed to ethanol did not metabolize CMFDA but appeared bright red due to PI internalization, indicating that here the membrane was permeabilized. We therefore conclude that the rapid increase in [Ca^2+^]_c _within the first 10 min of protein treatment is not the result of uncontrolled Ca^2+ ^influx due to plasma membrane permeabilization.

### The calcium chelator BAPTA abrogates the AFP_NN5353_-induced calcium signature

**Figure 4:**
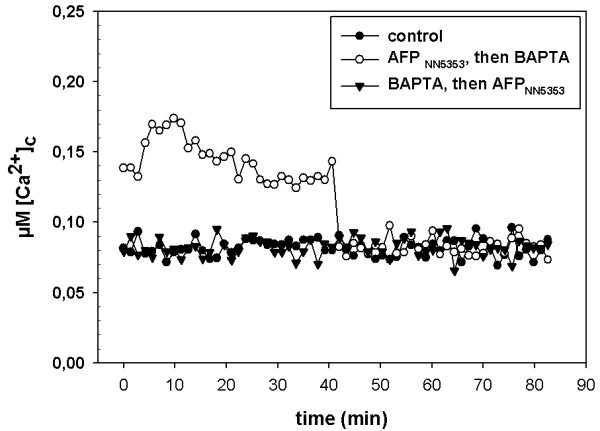
**Effect of the extracellular chelator BAPTA on the AFP_NN5353 _induced [Ca^2+^]_c _resting level**. 10 mM BAPTA (final conc.) were applied 40 min before or 40 min after treatment with 20 μg/ml AFP_NN5353_. Samples without supplements were used as controls. SD (n = 6) was less than 10% of the values presented.

The increased [Ca^2+^]_c _in response to AFP_NN5353 _treatment could originate from extracellular and/or from intracellular Ca^2+ ^stores, such as mitochondria, vacuoles, endoplasmic reticulum or the Golgi apparatus. To discriminate between the extracellular and intracellular source of the [Ca^2+^]_c _increase, we tested the influence of the Ca^2+^-selective membrane impermeable chelator BAPTA. On its own, BAPTA did not influence the resting level of [Ca^2+^]_c _in twelve h old *A. niger *cultures (Figure 4). However, a pretreatment of the samples with 10 mM BAPTA before the addition of AFP_NN5353 _inhibited the protein-specific increase in [Ca^2+^]_c _resting level (Figure 4). Interestingly, the elevated [Ca^2+^]_c _in response to a 40 min AFP_NN5353_-treatment dropped to the resting level immediately after the addition of 10 mM BAPTA (Figure 4), indicating that the AFP_NN5353_-induced elevation of the [Ca^2+^]_c _resting level requires the continuous influx of extracellular Ca^2+ ^and eventually results in loss of [Ca^2+^]_c _homeostasis.

### Extracellular calcium ameliorates the AFP_NN5353_-induced rise in [Ca^2+^]_c_

To decipher the observation that high external CaCl_2 _concentrations counteracted AFP_NN5353 _toxicity (Table 3), we monitored the effect of externally added Ca^2+ ^on the AFP_NN5353_-induced Ca^2+ ^signature. To this end, *A. niger *germlings were preincubated with 20 mM CaCl_2 _for 10 min before 20 μg/ml AFP_NN5353 _was added and the changes in the [Ca^2+^]_c _resting level were monitored over a time course of 60 min. This treatment resulted in a less pronounced rise of the [Ca^2+^]_c _resting level compared to samples without preincubation with CaCl_2_. In contrast, the presence of 20 mM CaCl_2 _alone had no major effect on the intracellular [Ca^2+^]_c _resting level which resembled that of the control without AFP_NN5353 _(data not shown). The values of the [Ca^2+^]_c _resting levels of the last 10 min (50 to 60 min) measurement of AFP_NN5353 _treatment in the presence or absence of high Ca^2+ ^concentration (20 mM versus 0.7 mM) are summarized in Table 4. The average of the [Ca^2+^]_c _of the controls which were not exposed to AFP_NN5353 _was 0.039 μM in the presence of 0.7 μM CaCl_2 _(standard condition) and 0.062 μM in the presence of 20 mM CaCl_2_. When AFP_NN5353 _was added, there was no significant elevation of the [Ca^2+^]_c _in high-Ca^2+ ^medium (20 mM) (0.057 μM) whereas the [Ca^2+^]_c _rised to 0.146 μM at standard CaCl_2 _concentration (0.7 mM). These results suggest that Ca^2+ ^externally added prior to the addition of AFP_NN5353 _counteracts the AFP_NN5353 _induced perturbation of the [Ca^2+^]_c _and growth inhibitory effect, at least partly, by controlling the [Ca^2+^]_c _resting level.

**Table 4:** The effect of high external CaCl_2 _concentration on the AFP_NN5353 _induced Ca^2+ ^signature in response to AFP_NN5353_.

**[CaCl**_**2**_] in Vogels*	**0 μg/ml AFP**_**NN5353**_	**20 μg/ml AFP**_**NN5353**_
0.7 mM	0.039 (_SD _± 0.001)	0.146 (_SD _± 0.009)
20 mM	0.062 (_SD _± 0.003)	0.057 (_SD _± 0.004)

Twelve h old germlings were preincubated with 20 mM CaCl_2 _for 10 min before exposure to AFP_NN5353_. Values represent the average μM concentration of [Ca^2+^]_c _within the last 10 min (50-60 min) of measurement.

### AFP_NN5353 _decreases the amplitude of the [Ca^2+^]_c _response to mechanical perturbation in *A. niger*

**Figure 5:**
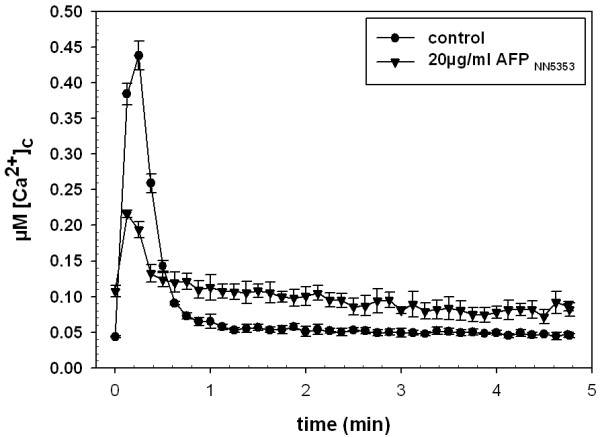
**Effects of AFP_NN5353 _on the [Ca^2+^]_c _response to mechanical perturbation**. Twelve h old *A. niger *cultures were treated with 20 μg/ml AFP_NN5353 _for 60 min before stimulation by mechanical perturbation (addition of 100 μl Vogels medium). The [Ca^2+^]_c _signature was monitored for 5 min. Values represent the average of six samples.

It is known that a range of external stimuli transiently increase [Ca^2+^]_c _levels in *Aspergilli *and other fungi [31,32]. One of these physiological stimuli is mechanical perturbation, which is achieved by the rapid injection of isotonic medium into the test system. This stimulus results in a unique Ca^2+ ^signature, likely involving different components of the Ca^2+^-signalling and Ca^2+ ^homeostatic machinery. Changes in this specific Ca^2+ ^signature in the presence of compounds, such as AFP_NN5353_, can give insights into the mode of action of these compounds. In our study, twelve h old cultures of *A. niger *were pre-incubated with AFP_NN5353 _for 60 min and thereafter subjected to mechanical perturbation (rapid injection of 100 μl Vogels medium). The resulting Ca^2+ ^signature, including [Ca^2+^]_c _resting level, kinetics and amplitude, were determined and compared with controls that were not exposed to the protein but also subjected to mechanical perturbation. As shown in Figure 5, AFP_NN5353 _provoked a less pronounced [Ca^2+^]_c _amplitude; however, the [Ca^2+^]_c _level remained elevated even after the stimulus specific response had stopped.

### AFP_NN5353 _binding and uptake are essential for protein toxicity in *A. nidulans*

To understand the function of antifungal proteins, the identification of the site of action in target organisms is crucial. So far, controversial reports exist of the localization of the homologous *A. giganteus *AFP protein. AFP has been detected to bind to outer layers, e.g. the cell wall or the plasma membrane of sensitive fungi [20,21] and a time- and concentration-dependent intracellular localization was reported [20]. In another study, Alexa-labelled AFP was shown to be internalized by the fungal cell and to localize to the nucleus [33].

**Figure 6:**
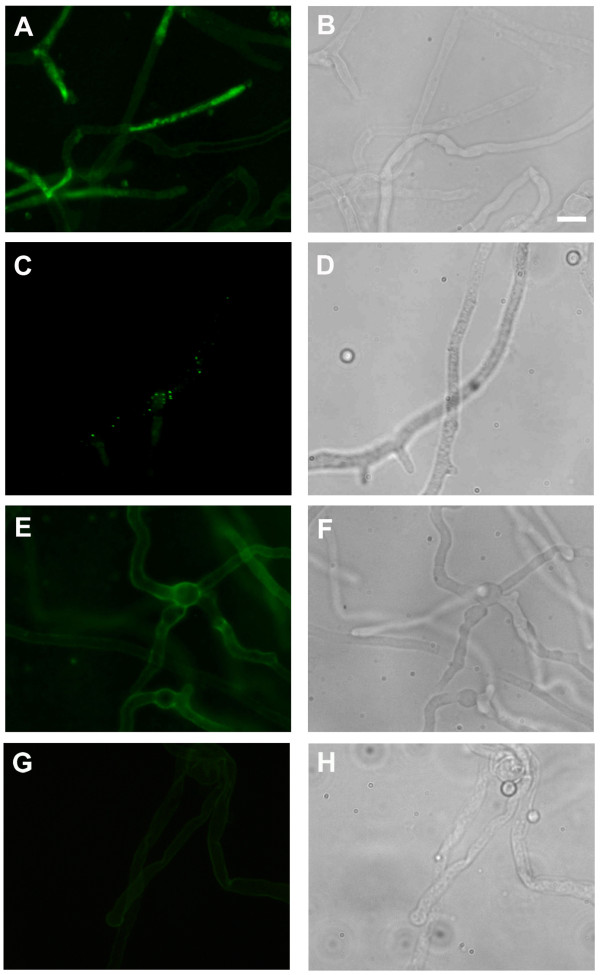
**Indirect immunofluorescence staining of *A. nidulans *with rabbit anti-AFP_NN5353 _antibody**. Fungi were incubated with 0.2 μg/ml AFP_NN5353 _**(A, E, G) **or without antifungal protein **(C)**. 20 μg/ml latrunculin B **(E) **and 10 mM Ca^2+ ^**(G) **significantly reduced protein uptake. **(B, D, F, H) **are the respective light microscopic images of **(A, C, E, G)**. Scale bar 10 μm.

To dissect the uptake and localization of AFP_NN5353_, we performed indirect immunofluorescence staining with *A. nidulans *wild type exposed to a sublethal concentration of AFP_NN5353 _(0.2 μg/ml). We applied a protein amount below the toxic concentration for hyphae to maintain the cellular structure and to avoid apoptotic cell disruption [34]. Our study revealed that the protein was internalized after 90 min of incubation, mostly in hyphal tips, but also within hyphal segments (Figure 6A, B). The protein seemed not to localize to cell compartments, but was distributed in the cytoplasm. Similar results were obtained with *A. niger *wild type (data not shown). Control experiments proved the specificity of the intracellular immunofluorescent signals: no intracellular fluorescent signals were detected in samples where either AFP_NN5353 _(Figure 6C, D) or the primary antibody or the secondary antibody was omitted (data not shown).

To analyse the AFP_NN5353 _localization in more detail, *A. nidulans *was incubated with AFP_NN5353 _in the presence of latrunculin B, a potent inhibitor of actin polymerization and endocytosis [35-37]. At low latrunculin B concentrations (5 μg/ml), protein uptake was severely reduced compared to the positive control without latrunculin B (data not shown), whereas 20 μg latrunculin B/ml completely inhibited the uptake of 0.2 μg/ml AFP_NN5353_. The solvent of latrunculin B, DMSO, had no adverse effect on protein uptake (data not shown). This indicates that AFP_NN5353 _enters the *A. nidulans *cells by an endocytotic mechanism (Figure 6E, F).

Based on our observation that Ca^2+ ^ions antagonize the growth inhibitory activity of AFP_NN5353_, we questioned whether Ca^2+ ^prevents actin-mediated internalisation of the antifungal protein. Indeed, the presence of 10 mM CaCl_2 _inhibited protein uptake (Figure 6G, H). Most interestingly, no specific fluorescent signals were detectable in *M. circinelloides *when treated with up to 500 μg/ml of antifungal protein (data not shown), indicating that AFP_NN5353 _does not bind to insensitive strains.

## Discussion

In this study we provide important insights into the mechanistic basis of AFP_NN5353_, a AFP homologous protein.

Species specificity tests revealed that AFP_NN5353 _is active against a broad range of filamentous fungi, including human and plant pathogens. Although the proteins AFP_NN5353 _and AFP are almost identical and show a similar toxicity, MICs for AFP_NN5353 _differed slightly from those reported for AFP [21]. We attribute this discrepancy to differences in the experimental setups, e.g. fungal strains, medium composition, conidial inoculum, incubation times, cultivation temperature etc., rather than to the differences in the primary sequence of both proteins.

It has been reported that the closely related AFP protein interfered with cell wall synthesis [10] and our finding that the osmotic stabilizer sorbitol neutralized AFP_NN5353 _toxicity further corroborated this assumption. Two *A. nidulans *mutants, the conditional *alcA-*PkcA and the *mpkA *deletion mutant showed a hypersensitive phenotype when exposed to AFP_NN5353_. This is in agreement to the reported function of cell wall stressing agents, such as CFW or caffeine in *S. cerevisiae *and *A. nidulans *[9,16,24,26,38,39] and to the *Penicillium *antifungal protein PAF [9]. Importantly, Mpk function is essential for CWIP activation in both, unicellular and filamentous fungi [10,16,40] and triggers the activation of the transcription factors Rlm1p and SBF which regulate the expression of cell cycle regulated genes and genes involved in the synthesis and remodelling of the fungal cell wall in *S. cerevisiae *[41,42]. Similarly, RlmA dependent induction of the expression of the *ags *gene was also reported for aspergilli [25]. Importantly, the activation of the CWIP can occur in a RhoA-dependent, e.g. with CFW [9,43], or RhoA-independent way, the latter proved for PAF and caffeine [9,16] and for AFP_NN5353 _(this study). As proposed by [28] the dominant *rhoA^E40I ^*allele suffers from a perturbation of its GAP binding domain and downstream effectors of Rho-GAP might be disturbed. Therefore, we hypothesize that Rho-GAP targets might be involved in the toxicity of AFP_NN5353 _similarly to the mode of action of the *P. chrysogenum *PAF [9]. Our assumption of the activation of the CWIP by AFP_NN5353 _was further strengthened by the fact, that AFP_NN5353 _treatment induced *agsA *expression in the *A. niger *reporter strain. This result was consistent with the activity of AFP and caspofungin [10], but differed to the function of PAF, where no CWIP activation and no induction of cell wall biosynthesis genes occurred [9].

Therefore, we conclude that AFP_NN5353 _triggers cell wall remodeling via Pkc/Mpk signalling. We further deduce from our data that similarities and differences exist in the molecular targets and the mode of action of antifungal proteins from filamentous fungi, e.g. AFP_NN5353 _and PAF - despite their homology. This phenomenon was also reported for other closely related antifungal proteins, such as the plant defensins MsDef1 and MtDef4 from *Medicago spp*. [44].

Apart from the activation of the CWIP, the perturbation of the Ca^2+ ^homeostasis represents a major mechanistic function of antifungal proteins in sensitive fungi [17,18]. The intracellular Ca^2+ ^response to AFP_NN5353 _in *A. niger *reflected that of the *Penicillium *antifungal protein PAF in *N. crassa *[17]. The rapid and sustained increase of the [Ca^2+^]_c _resting level depended on a sustained influx of Ca^2+ ^ions from the external medium. Moreover, the AFP_NN5353 _induced changes in the Ca^2+ ^signature of mechanically perturbed *A. niger *cells further underlines the disruption of the Ca^2+ ^response and homeostasis by AFP_NN5353_. The addition of CaCl_2 _to the growth medium reduced the susceptibility of *A. niger *towards the antifungal protein and decreased the AFP_NN5353 _specific rise in the [Ca^2+^]_c _resting level. Both observations point towards an adaptive response which is mediated most probably via Ca^2+ ^signalling. First, high extracellular Ca^2+ ^concentrations trigger chitin synthesis in *A. niger *and thereby confer increased protection against antifungal proteins as shown for AFP [15]. Second, it primes the Ca^2+ ^homeostatic machinery to better maintain a low [Ca^2+^]_c _resting level when challenged with the antifungal protein, e.g. by (i) the increase of the activity of existing Ca^2+ ^pumps/transporters to counteract the AFP_NN5353_-specific intracellular Ca^2+ ^perturbation, or (ii) the modulation of the expression of Ca^2+ ^channels/pumps/exchangers [17]. The former hypothesis (i) might be supported by the observation that the addition of CaCl_2 _only 10 min before *A. niger *was challenged with AFP_NN5353 _restored the low [Ca^2+^]_c _resting level. However, the perturbation of the Ca^2+ ^homeostasis by a sustained elevation of the [Ca^2+^]_c _resting level indicates that *A. niger *is not able to restore the low [Ca^2+^]_c _resting level after exposure to AFP_NN5353 _and this might trigger programmed cell death (PCD) on the long term as it was shown to occur in *A. nidulans *in response to the *P. chrysogenum *PAF [34].

Since AFP was shown to cause membrane permeabilization [21], the influx of Ca^2+ ^might be due to changes in membrane permeability for this ion, if not the formation of pores. However, our staining experiments with CMFDA and PI exclude this possibility at least in the first 10 min of exposure to AFP_NN5353 _when the [Ca^2+^]_c _resting level reaches its maximum. This result is further corroborated by the fact that higher external concentrations of Ca^2+ ^reduced the AFP_NN5353 _specific rise in [Ca^2+^]_c _resting level which - in our opinion - would not occur with leaky membranes. However, we do not exclude changes in membrane permeability at longer exposure times to this antifungal protein and more studies are needed to answer this question.

Finally, we observed that the internalization of AFP_NN5353 _is characteristic for sensitive but not resistant moulds. A lack of binding of AFP_NN5353 _to insensitive fungi might point towards the absence or inaccessibility of a putative interacting molecule at the cell surface. AFP_NN5353 _localized to the cytoplasm of target fungi only when actin filaments were formed. This is in agreement with the endocytotic uptake and intracellular localization of the *P. chrysogenum *antifungal protein PAF in sensitive filamentous fungi [14,45]. Importantly, we observed that AFP_NN5353 _was internalized by hyphae even under sub-inhibitory concentrations (0.2 μg/ml for *A. nidulans*) which suggests that a threshold concentration is required to cause severe growth defects in target fungi.

The presence of high concentrations of extracellular Ca^2+ ^counteracted AFP_NN5353 _uptake. This finding parallels well with the report of [20] that the presence of cations, such as Ca^2+^, interfered with the binding of AFP to the surface of *F. oxysporum *and with our observations made with the *Penicillium *PAF (unpublished data). One possible explanation might be that extracellular Ca^2+ ^ions compete with AFP_NN5353 _for the same molecular target on the fungal surface which might represent a first binding receptor or even a "gate" for protein uptake [20,21] or, alternatively, that the interacting target is repressed under these conditions [17]. An additional explanation might be that the primary cell-surface localized AFP_NN5353 _target might be masked due to a Ca^2+^-dependent stimulation of chitin synthesis and cell wall remodeling as recently observed for AFP in *A. niger *[15]. This further suggests that the activation of the CWIP and the *agsA *induction does not mediate sufficient resistance to survive the toxic effects of AFP_NN5353_. Instead, according to the "damage-response framework of AFP-fungal interactions" [15], the chitin response might represent the better strategy for fungi to survive the antifungal attack.

## Conclusions

**Figure 7:**
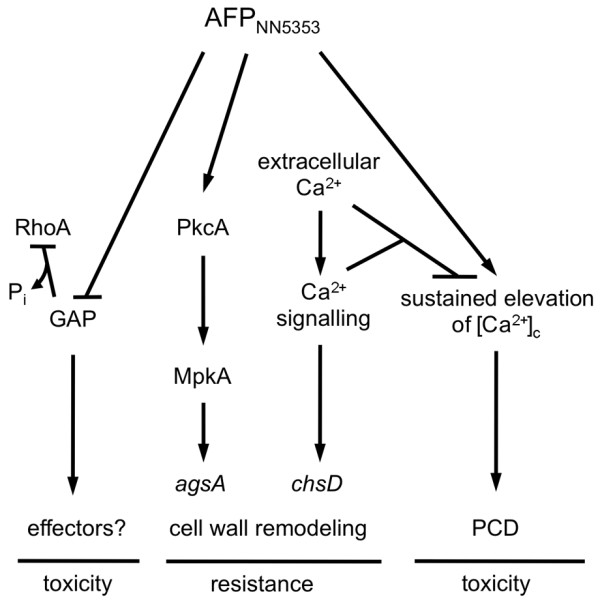
**Tentative model of the mechanistic function of the *A. giganteus *antifungal protein AFP_NN5353 _on *Aspergillus sp***. The response against AFP_NN5353 _attack is mediated via PkcA/MpkA signalling and results in increased *agsA *transcription. However, the activity of the CWIP occurs independently from RhoA and so far unidentified RhoA-GAP effector molecules might contribute to the AFP_NN5353 _toxicity. Furthermore, AFP_NN5353 _leads to an immediate and significant increase of the [Ca^2+^]_c _resting level in the cell. The sustained perturbation of the Ca^2+ ^homeostasis could lead to PCD [17,34]. The presence of elevated concentrations of extracellular Ca^2+ ^counteracts the toxic effects of AFP_NN5353 _and improves the resistance of the target organism by decreasing the elevated [Ca^2+^]_c _resting level. Whereas cell wall remodelling via CWIP seems to be insufficient to counteract AFP_NN5353 _activity, the fortification of the cell wall by the induction of *chsD *expression might represent an adequate response to increase resistance [15].

Based on the growth inhibitory activity, antifungal proteins like AFP_NN5353 _can be well considered as promising candidates for future antimycotic drug developments. However, for biotechnological exploitation, the detailed knowledge on the mode of action is demanded. Our study shows that the detrimental effects caused by the *A. giganteus *antifungal protein AFP_NN5353 _in sensitive target aspergilli are based on the interaction of this protein with more than one signalling pathway. In Figure 7, we present a tentative working model. The toxicity of AFP_NN5353 _is mediated via PkcA/MpkA signalling which occurs independently from RhoA. Instead, so far unidentified RhoA-GAP effector molecules might contribute to AFP_NN5353 _toxicity. The activation of the CWIP by AFP_NN5353 _induces the *agsA *gene expression which is, however, insufficient to counteract toxicity of the protein. Furthermore, AFP_NN5353 _leads to an immediate and significant increase of the [Ca^2+^]_c _resting level in the cell. This sustained perturbation of the Ca^2+ ^homeostasis could lead to PCD [17,34]. The presence of extracellular Ca^2+ ^neutralizes the toxic effects of AFP_NN5353 _and improves the resistance of the target organism possibly by decreasing the elevated [Ca^2+^]_c _resting level and stimulating the fortification of the cell wall by the induction of *chsD *expression as shown for AFP [15]. Further investigations are in progress to clarify how these pathways are interconnected and interfere with each other on the molecular level.

## Methods

### Strains, Media and Chemicals

Fungal strains used in this study are listed in Table 5. All strains were obtained from the culture collections FGSC, ATCC, CBS, from the Institute of Microbiology, Division of Systematics, Taxonomy and Evolutionary Biology at the Leopold Franzens University of Innsbruck, or the strain collection of the Department of Biotechnology, National Institute of Chemistry, Ljubljana, Slovenia. Unless otherwise stated, all fungi were grown in complete medium (CM) [19] with the respective supplements [28,38]. R153 and *alcA*-PkcA were grown in defined minimal medium (MM) according to [26]. Ca^2+ ^response experiments were performed in Vogels medium [46]. For experiments with CaCl_2 _supplementation, the KH_2_PO_4 _concentration of the culture media was reduced from 37 mM to 10 mM to avoid precipitation of supplemental Ca^2+ ^and these media were called CM* and Vogels*. Chemicals were purchased from Sigma. AFP_NN5353 _and polyconal rabbit anti-AFP_NN5353 _antibody were generous gifts from Mogens T. Hansen, Novozymes, Denmark. The antifungal protein was isolated from *A. giganteus *strain A3274 (CBS 526.65), purified and analyzed by HPLC as described in the patent application WO94/01459 [47].

**Table 5:** Fungal strains used in this study.

Strain	Relevant genotype	Source or reference
*A. flavus *ATCC 9643	wild type	ATCC
*A. fumigatus *ATCC 46645	wild type	ATCC
*A. giganteus *AG 090701	wild type isolate	Institute of Microbiology
*A. nidulans*		
FGSC A4	Glasgow wild type (veA^+^); velvet mutant	FGSC
R153	*wA2; pyroA4*	[26]
*alcA*-PkcA	*pyrG89::pyr4 alcA(p)::pkcAΔp*	[26]
GR5	*pyrG89; wA3; pyroA4*	[28]
RhoA^G14V^	GR5 + pGG2 (*rhoA*^G14V^) and pRG3AMA1 (co-transformation plasmid)	[28]
RhoA^E40I^	GR5 + pGG5 (*rhoA*^E40I^) and pRG3AMA1 (co-transformation plasmid)	[28]
Δ*mpkA*	Δ*mpkA*	[38]
*A. niger*		
CBS 120.49	wild type	CBS
A533	*cspA1, aeq*S, *amd*S^+ ^(pAEQS1-15)	[31]
RD6.47	P *agsA*::*h2b*::*egfp*::*Ttrpc*	[10]
*A. terreus *304	wild type isolate	Institute of Microbiology
*Botrytis cinerea *BC 080801	wild type isolate	Institute of Microbiology
*Fusarium oxysporum *FO 240901	wild type isolate	Institute of Microbiology
*F. sambucinum *FS 210901	wild type isolate	Institute of Microbiology
*Gliocladium roseum *GR 210901	wild type isolate	Institute of Microbiology
*M. circinelloides *MC 080801	wild type isolate	Institute of Microbiology
*M. genevensis *MG 080801	wild type isolate	Institute of Microbiology
*P. chrysogenum *ATCC 10002	wild type	ATCC
*Trichoderma koningii *TC 060901	wild type isolate	Institute of Microbiology
*Neuropsora crassa *FGSC 2489	wild type	FGSC

### Growth inhibition assays

Antifungal activity assays were performed in 96-well plates in CM or Vogels medium inoculated with 1 × 10^4 ^conidia/ml and supplemented with various concentrations of AFP_NN5353 _or with equivalent amounts of buffer (untreated controls). Fungal growth was monitored microscopically with an Olympus CK40 microscope equipped with a Zeiss MRc digital camera and the growth rates were determined spectrophotometrically as described previously [19]. Alternatively, 2 × 10^3 ^conidia were spotted in 5 μl aliquots on appropriately supplemented agar plates. The plates were then incubated at 37°C for up to 72 h. Every 24 h, the plates were photographed and the colony diameters were determined. All assays were performed as technical triplicates and biological duplicates.

### Analysis of the induction of the *agsA *expression by a GFP-based reporter system

The *A. niger *reporter strain RD6.47 carries the *agsA *promoter fused to a nucleus-targeted GFP (H2B::eGFP) [27]. Activation of the CWIP can be monitored by the increase in nuclear fluorescence. Analysis of the activation of the *agsA *promoter by 10-100 μg/ml AFP_NN5353 _was performed as described in [10]. As a positive control, caspofungin at a concentration of 10 μg/ml was used. Fluorescence images were taken from coverslips observed with an Axioplan 2 microscope (Zeiss) equipped with a Sony DKC-5000 digital camera.

### Fluorescence staining

#### Indirect immunofluorescence staining

*A. nidulans *was grown over night on glass cover slips at 30°C in CM. They were further incubated for 90 min in the presence or absence (controls) of 0.2 μg/ml AFP_NN5353_. The samples were stained as described previously [14] and incubated with rabbit-anti-AFP_NN5353 _antibody (1:2.500, Novozymes, Denmark) for at least 60 min. Immunocomplexes were detected with FITC-conjugated swine-anti-rabbit IgG (1:40, DAKO, Germany). All samples were embedded in Vectashield mounting medium (Vector Laboratories, Burlingame, USA). Microscopy was done with a Zeiss Axioplan fluorescence microscope or a Zeiss confocal laser scanning microscope as described in [14].

For incubation with latrunculin B (Sigma, Austria), samples were treated with 0.2 μg/ml AFP_NN5353 _and 10 μg/ml latrunculin B for 80 min. As a control, samples were treated with DMSO to exclude artifacts evoked by the dissolvent of latrunculin B.

For detection of AFP_NN5353 _in the presence of elevated concentrations of CaCl_2_, fungi were grown in CM* medium and then treated with 0.2 μg/ml AFP_NN5353 _in the presence of 10 mM CaCl_2 _for 90 min.

#### Analysis of membrane permeabilization and cell viability

To determine if AFP_NN5353 _permeabilized the plasma membrane of *A. niger *germlings, we used a combination of propidium iodide (PI) and fluorescein diacetate (cell tracker, CMFDA green, Invitrogen) according to [48]. Twelve h old *A. niger *germlings were grown in Vogels medium and pretreated with the two dyes (final conc. 5 μg/ml each) for 15 min before AFP_NN5353 _was added to a final concentration of 20 μg/ml. Samples without AFP_NN5353 _served as controls for positive CMFDA staining, while ethanol (70%) was used to permeabilize the membrane for positive PI staining.

#### Analysis of the calcium response to AFP_NN5353 _application

10^5 ^conidia/ml of the *A. niger *strain A533 expressing codon optimized aequorin were grown in Vogels* medium containing 10 μM coelenterazine (Biosynth, Switzerland) at 30°C for twelve h in the dark. The [Ca^2+^]_c _resting level and mechanical perturbation experiments and the calibration of [Ca^2+^]_c _were performed as described in [17].

## Authors' contributions

UB carried out the growth inhibition assays, the indirect immunofluorescence stainings, the Ca^2+ ^measurements and the calculations to convert the luminescence units into the [Ca^2+^]_c _levels. She also performed the statistical analysis and helped to draft the manuscript. MB contributed the *A. niger *A533 strain, helped with the Ca^2+ ^measurements and participated in the design of the study. AE contributed to the indirect immunofluorescence stainings. VM contributed the *A. niger *RD6.47 strain and performed the *agsA *induction assays. FM conceived of the study, participated in its design and coordination and drafted the manuscript. All authors read and approved the final manuscript.

## Supplementary Material

Additional file 1 **The expression of nucleus-targeted GFP under the control of the *agsA *promoter in *A. niger *in response to cell wall interfering substances**. Differential interfering contrast images and corresponding fluorescence images of *A. niger *RD6.47 indicate the expression of a nucleus-targeted GFP under the control of the *A. niger agsA *promoter. Five h old germlings were **(A) **left untreated (negative control), **(B) **treated with 50 μg/ml AFP_NN5353 _and **(C) **with 10 μg/ml caspofungin (positive control) as described in Materials and Methods. Scale bar, 20 μm. Click here for file

Additional file 2 **Viability staining of *A. niger *germlings after AFP_NN5353 _exposure**. Twelve h old *A. niger *germlings were stained with fluorescein diacetate (CMFDA, middle pannels) and propidium iodide (right pannels). The left panels show the respective light micrographs. All samples were pretreated with the dyes for 15 min before 20 μg/ml AFP_NN5353 _was added **(B)**. Controls remained untreated **(A) **or were exposed to 70% ethanol **(C)**. Scale bar, 50 μm. Click here for file
