# Supplementary figures and images for: The Aspergillus giganteus antifungal protein AFPNN5353 activates the cell wall integrity pathway and perturbs calcium homeostasis
Source: BMC Microbiol. 2011 Sep 23;11:209. doi: 10.1186/1471-2180-11-209 (PMC3197501; doi:10.1186/1471-2180-11-209)

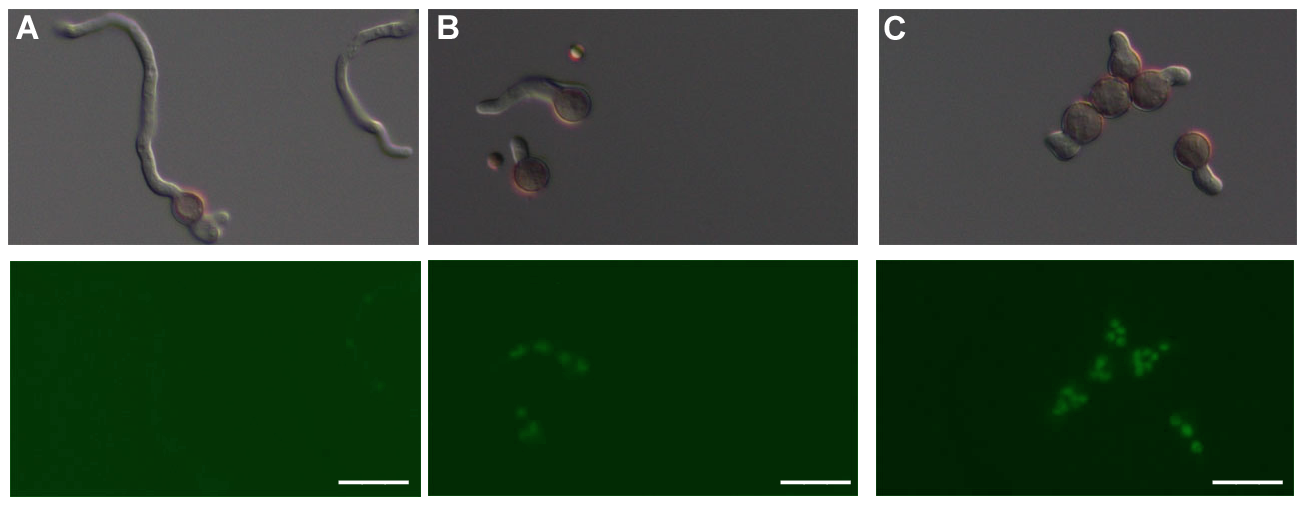

Supplement: Additional file 1 — The expression of nucleus-targeted GFP under the control of the agsA promoter in A. niger in response to cell wall interfering substances. Differential interfering contrast images and corresponding fluorescence images of A. niger RD6.47 indicate the expression of a nucleus-targeted GFP under the control of the A. niger agsA promoter. Five h old germlings were (A) left untreated (negative control), (B) treated with 50 μg/ml AFPNN5353 and (C) with 10 μg/ml caspofungin (positive control) as described in Materials and Methods. Scale bar, 20 μm. [file 1471-2180-11-209-S1.TIFF]

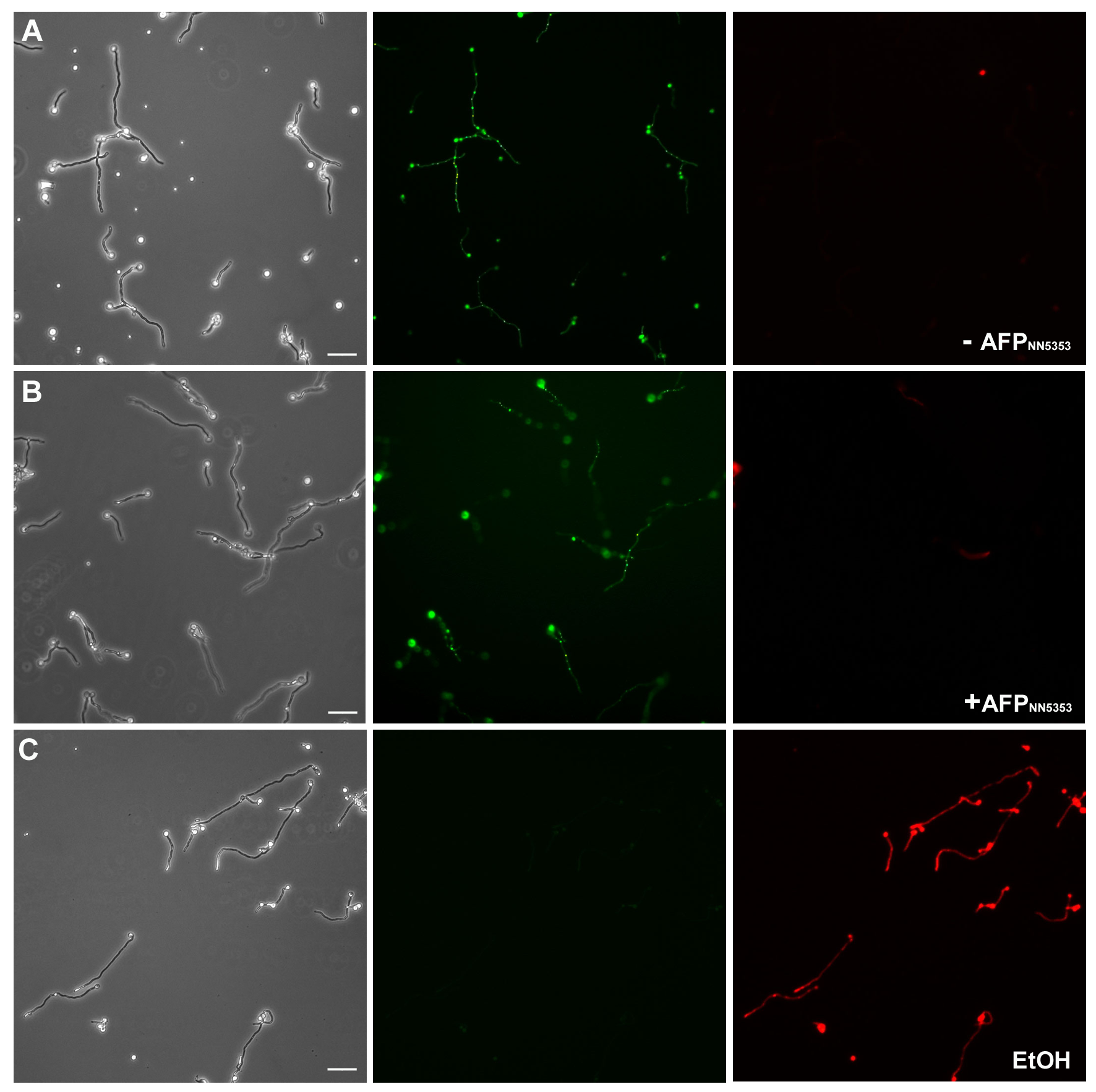

Supplement: Additional file 2 — Viability staining of A. niger germlings after AFPNN5353 exposure. Twelve h old A. niger germlings were stained with fluorescein diacetate (CMFDA, middle pannels) and propidium iodide (right pannels). The left panels show the respective light micrographs. All samples were pretreated with the dyes for 15 min before 20 μg/ml AFPNN5353 was added (B). Controls remained untreated (A) or were exposed to 70% ethanol (C). Scale bar, 50 μm. [file 1471-2180-11-209-S2.TIFF]
